# Generating conditional gene knockouts in *Plasmodium* – a toolkit to produce stable DiCre recombinase-expressing parasite lines using CRISPR/Cas9

**DOI:** 10.1038/s41598-017-03984-3

**Published:** 2017-06-20

**Authors:** Ellen Knuepfer, Marta Napiorkowska, Christiaan van Ooij, Anthony A. Holder

**Affiliations:** 1 Malaria Parasitology Laboratory, The Francis Crick Institute, 1 Midland Road, London, NW1 1AT United Kingdom; 2 Malaria Biochemistry Laboratory, The Francis Crick Institute, 1 Midland Road, London, NW1 1AT United Kingdom; 3 Department of Biosystems Science and Engineering, ETH Zurich, Mattenstrasse 26, Basel, 4058 Switzerland

## Abstract

Successful establishment of CRISPR/Cas9 genome editing technology in *Plasmodium* spp. has provided a powerful tool to transform *Plasmodium falciparum* into a genetically more tractable organism. Conditional gene regulation approaches are required to study the function of gene products critical for growth and erythrocyte invasion of blood stage parasites. Here we employ CRISPR/Cas9 to facilitate use of the dimerisable Cre-recombinase (DiCre) that is frequently used to mediate the excision and loss of *loxP*-flanked DNA sequences in a rapamycin controlled manner. We describe novel CRISPR/Cas9 transfection plasmids and approaches for the speedy, stable and marker-free introduction of transgenes encoding the DiCre recombinase into genomic loci dispensable for blood stage development. Together these plasmids form a toolkit that will allow the rapid generation of transgenic DiCre-expressing *P. falciparum* lines in any genetic background. Furthermore, the newly developed 3D7-derived parasite lines, constitutively and stably expressing DiCre, generated using this toolkit will prove useful for the analysis of gene products. Lastly, we introduce an improved treatment protocol that uses a lower rapamycin concentration and shorter treatment times, leading to *loxP*-guided recombination with close to 100% efficiency within the same replication cycle.

## Introduction

Malaria is one of the most significant infectious diseases of people in low income countries, with an estimated 212 million cases in 2015 causing around half a million deaths. 99% of these deaths occur as a consequence of *P. falciparum* infections, in particular affecting children below the age of five in Africa^1^. The fast spread of drug resistance^2^ and the limited efficacy of current vaccines^3^ highlight the pressing need to identify new targets for therapeutic intervention. In 2002, the genome sequence of the 3D7 line of *P. falciparum* was published^4^. It encodes roughly 5400 genes, half of which have no assigned function, in part due to the limited sequence similarity with genes outside of the *Plasmodium* genus.

Recently, the CRISPR/Cas9 (clustered regularly interspaced short palindromic repeats/ CRISPR-associated protein 9) genome editing system has been successfully adapted for use in *Plasmodium* spp., revolutionising genetic engineering in these parasites^5–7^.

The parasite is haploid for much of its life cycle, including during the asexual erythrocytic cycle, requiring conditional genetic modification systems to study essential gene functions. Such systems allowing for control over mRNA abundance and stability, translation, and protein turnover have been developed but these approaches often result in only partial depletion of the gene product^8–11^. As a consequence, results from these studies can be difficult to interpret. A recent report describes protein mislocalisation induced by addition of a small molecule as a new conditional system to study protein functions in *P. falciparum* ^12^, allowing the investigation of proteins that can be modified at the N terminus or C terminus and are not secreted. In other organisms site-specific recombinases are widely used to modify genomes, for example the total removal of a target gene or an important part thereof. The use of Cre- and FLP-recombinase has been described in *Plasmodium spp*.^13–16^. The inducible Cre recombinase (DiCre) established for use in *P. falciparum* uses the rapamycin-binding FKBP12 and FRB proteins to dimerise the two enzyme halves^17^; this system was first established in Apicomplexan parasites in *Toxoplasma gondii* ^18^ and then adapted successfully to *P. falciparum* ^13, 14^. This site–specific recombinase recognises short, 34 bp sequences called *loxP* sites and catalyses the excision or inversion of the floxed (flanked by *loxP*) DNA segment. *Lo*x*P* sites can be introduced silently within the ORF in the form of an artificial intron (*loxPint*)^19^, or can be introduced into endogenous introns or into UTRs. Conditional excision of DNA sequences flanked by *loxP* sites can be designed such that in addition to removal of DNA sequences, domain swaps, introduction of point mutations and fusions to epitope tags can be generated.

The DiCre system has already been used to study the functions of gene products suspected to be essential for asexual blood stage development. Conditional knockouts of genes coding for MSP1, AMA1, RIPR, CyRPA, and PFA0210c have been generated, showing severe growth impairment that could not have been discovered using other methods^14, 20–22^.

Different systems have been developed to introduce DiCre into *P. falciparum*, including integration of DiCre cassettes into the chromosome and expression of the genes from an episome. Excision levels vary greatly between the systems, ranging from 50–98%^13, 14, 20, 21^. The integrated version of the system in the 3D7-derived 1G5DC parasite line tends to provide higher excision levels, but requires experimentation in that particular genetic background. In addition the 1G5DC line is subject to loss of the DiCre cassettes due to genetic reversion as the initial introduction of the DiCre cassettes has been generated through single cross-over recombination^23^. The episome-based expression of DiCre allows the DiCre system to be introduced in different parasite lines, but often provides lower excision levels.

Here we describe a toolkit of CRISPR/Cas9 and rescue plasmids to enable the quick, stable and marker-free generation of DiCre-expressing *P. falciparum* parasites. We demonstrate this by inserting DiCre recombinase into two loci, *p230p* and *pfs47*, which are dispensable for blood stage development as well as for infectivity to mosquitoes. In addition to the generation of the new DiCre recombinase-expressing parasite lines we have optimised the use of rapamycin for induction of Cre-recombinase activity. Lower concentrations of rapamycin for shorter exposure periods resulted in close to 100% excision within the same replication cycle. Using this toolkit to generate DiCre recombinase-expressing parasite lines will broaden the use of this conditional system, providing unrivalled levels of gene regulation in *Plasmodium* well beyond the 3D7 parasite line.

## Results

### Optimisation of rapamycin use to induce DiCre-mediated recombination activity

The rapamycin-inducible DiCre recombinase system has been used successfully for site-specific recombination in *Plasmodium* spp., resulting in deletion of whole or partial ORFs or UTRs^13, 14, 19–21^. In these studies, parasite cultures were treated with 100 nM rapamycin for a minimum of four hours and high levels of excision of floxed DNA sequence were reported. However, both rapamycin and the carrier, DMSO, can negatively affect parasite growth at higher concentrations. Using an already established floxed *PFA0210c* (PF3D7_0104200) line^22^, generated in the DiCre-expressing parasite line 1G5DC^13^, we set out to establish the lowest concentration of rapamycin required to achieve maximum excision levels.

**Figure 1:**
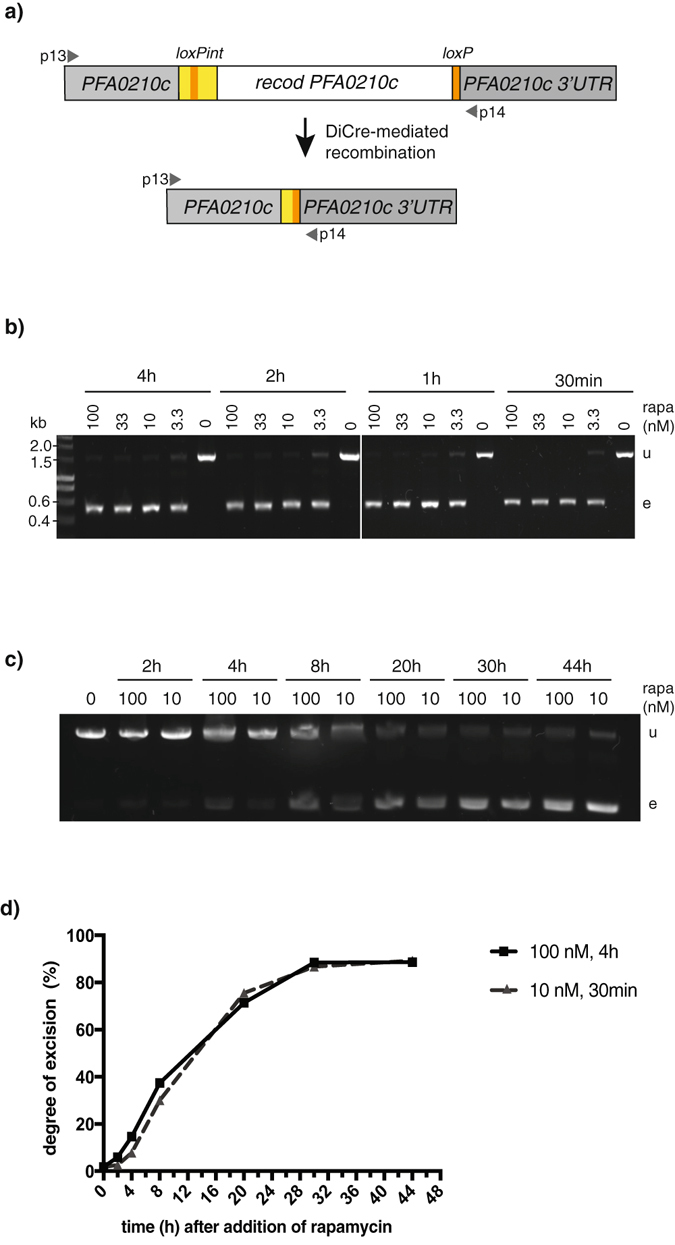
Optimisation of rapamycin-induced recombination. (**a**) Parasites containing a modified *PFA0210c* ^22^ were used to test DiCre-mediated recombination efficiency. For this a recodonised version of part of the ORF of *PFA0210c* was flanked by a *loxPint* sequence upstream and a *loxP* sequence downstream located within the endogenous locus on chromosome 1 in the 1G5DC parasite line. (**b**) The efficiency of excision was tested whilst altering the concentration and length of exposure to rapamycin (rapa). Either DMSO alone (0 nM) or increasing concentrations (in nM) of rapamycin were added to transgenic *PFA0210c-loxP* parasites as indicated for 4, 2, 1 h or 30 min. The degree of recombination was determined by PCR using primers 13 and 14 depicted as arrows in panel a). A lack of recombination and no excision resulted in a PCR product of 1580 bp (u) whereas successful excision resulted in a 562 bp product (**e**). The sizes of DNA markers (in kb) are depicted on the left. (**c**) The timing of excision was determined following addition of either 100 nM rapamycin for 4 h (100) or 10 nM rapamycin for 30 min (10) at very early ring stages. Parasites were harvested 2, 4, 8, 20, 30 or 44 h after the onset of treatment. Genomic DNA was extracted from saponin-treated parasite pellets and excision was determined by PCR as described in panel b. (**d**) Graph depicting the degree of excision (in %) over time elapsed after start of rapamycin treatment. For this graph, the data in panel c) was semi-quantitatively analysed. Pixel intensities of stained DNA bands were measured using ImageJ and the ratio of excised vs. unexcised was plotted against time after rapamycin addition, comparing two treatment methods (100 nM rapamycin for 4 h and 10 nM rapamycin for 30 min).

We treated *PFA0210c-loxP* parasites at early ring stage with 0, 3.3, 10, 33 or 100 nM rapamycin for between 30 min and 4 h and isolated genomic DNA 38–40 h later. PCR amplification of the *PFA0210c-loxP* locus revealed successful excision, as indicated by the decrease in size of the PCR product from 1580 bp to 562 bp, for all rapamycin concentrations of 10 nM and higher (Fig. 1a, b). Only in parasites treated with the lowest concentration of rapamycin (3.3 nM) did we detect a significant level of the unexcised locus. No difference was detected between treatment for 30 min and 4 h (Fig. 1b). We conclude that treatment with 10 nM rapamycin for 30 min, the shortest treatment period tested, is sufficient to induce maximum excision.

Comparing the previously used standard treatment to our newly determined minimal rapamycin exposure to achieve maximal DiCre recombinase activity, we examined recombination during the course of the lifecycle on parasites treated with 10 nM rapamycin for 30 minutes or 100 nM rapamycin for four hours. We determined the level of excision at 6 time points in one ~48-hour replication cycle (Fig. 1c,d). First, we observed no obvious difference in the degree of excision between the two treatment conditions at any time point. Second, a substantial level of excision was detected 20 h after the start of rapamycin treatment, with the maximum level achieved after 30 h. This result is in agreement with previously published data^14, 17^ and indicates that rapamycin-induced DiCre-mediated recombination is a slow process even if the formation of the Cre recombinase appears to be fast, as excision continues even after the removal of rapamycin.

### New CRISPR/Cas9 plasmids for stable insertion of DiCre recombinase into the *Plasmodium falciparum* genome

The previously available DiCre-expressing parasite line, 1G5DC, has proven to be an excellent tool to generate conditional gene modifications. However, this parasite line was generated by inserting the DiCre cassettes via single homologous recombination within the *SERA* locus on chromosome 2, resulting in duplication of 940 bp of *SERA5* DNA sequence^13^. We and others have detected occasional spontaneous reversion in these parasites to the wild type *SERA5* locus, resulting in the loss of the DiCre recombinase^23^ (Collins and Blackman, personal communication). To overcome this issue we applied a double homologous recombination approach using CRISPR/Cas9 gene editing to insert the DiCre cassette into one of two loci, *p230p* and *pfs47*, which have been validated as playing no role in the growth rate of *P. falciparum* asexual blood stages, or gametocyte production^24, 25^.

To insert the transgenes into a defined locus in the *P. falciparum* genome we used the CRISPR/Cas9 DNA editing tool. SpCas9 nuclease derived from *Streptococcus pyogenes* is targeted to a defined position in the genome by a single guide RNA, resulting in a DNA double-stranded break (DSB). DSB are normally lethal events unless repaired. *Plasmodium* parasites are deficient in DNA repair mechanisms such as canonical non-homologous end joining (C-NHEJ); instead the preferred repair mechanism is homologous recombination^26^. For this we provide a rescue plasmid containing DNA segments homologous in sequence to the target DNA close to the DSB site. Transfection of parasites with the CRISPR/Cas9 and the rescue plasmid results in exchange of DNA in the endogenous locus with a transgene flanked by the homologous DNA regions. Unlike single homologous recombination the vector backbone is not inserted into the chromosome and no duplication of homologous DNA sequences occurs in the genome.

**Figure 2:**
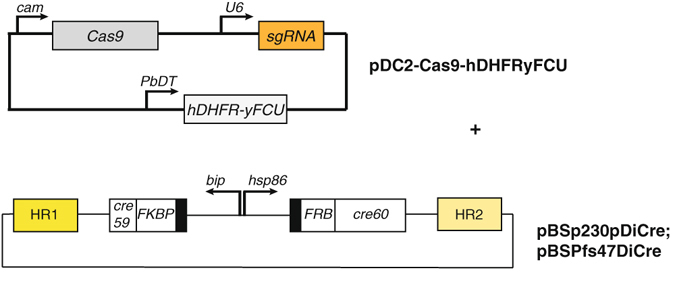
Toolkit plasmids for efficient integration of DiCre recombinase into *p230p* or *pfs47* loci of the *P. falciparum* genome. The CRISPR-Cas9 plasmid used here, pDC2-Cas9-hDHFRyFCU (derived from pDC2-Cas9-U6-h*dhfr* ^27^) carries the SpCas9 nuclease gene and a single guide RNA cassette driven by a short U6 promoter and the h*dhfr*-y*fcu* fusion gene for selection. The rescue plasmids (pBSp230pDiCre and pBSPfs47DiCre) contain the genes for the split Cre-recombinase fused to FRB and FKBP12 respectively, flanked by regions of homology from the *p230p* or the *pfs47* locus of *P. falciparum*. More detailed vector maps are available in Supplementary Fig. S2.

Our CRISPR/Cas9 gene editing tool uses two plasmids that are co-transfected (Fig. 2). The first plasmid, pDC2-Cas9-hDHFRyFCU, encodes the SpCas9 nuclease and the single guide RNA and is derived from pDC2-Cas9-U6-h*dhfr* ^27^. It was modified to contain a fusion of the positive selectable marker hDHFR (human dihydrofolate reductase) and the negative selectable marker yFCU (yeast cytosine deaminase/uridyl phosphoribosyl transferase) (Supplementary Fig. S1). For this, we amplified the h*dhfr*-y*fcu* cassette from pL0034^28^ using overlap extension PCR to destroy any BbsI restriction sites within the sequence. BbsI was used to insert the annealed guide RNA into the U6 promoter-terminator cassette resulting in seamless transcription of the single guide RNA. We reasoned that negative selection is desirable to select for parasites that have lost the CRISPR/Cas9 plasmid containing the positive selection marker. The second plasmid is a rescue plasmid, and as in Lu *et al*.^29^, it contains no selectable marker, allowing for marker-free modifications. In this case it is a pBluescript-derived plasmid carrying the complete expression cassettes coding for the two polypeptides forming the DiCre-recombinase. These two transgene cassettes were flanked by homology sequences of 500 bp to 976 bp, giving rise to pBSp230pDiCre and pBSPfs47DiCre (Supplementary Fig. S2).

The sequences used for homologous recombination in *pfs47* and *p230p* are based on the *P. falciparum* 3D7 line. However, to make this toolkit universally suitable for generating DiCre-expressing parasites from any *P. falciparum* line, we aimed to select the most conserved DNA sequences as homology regions (HR), which have to be in close proximity to the site in which the DSB is induced. *Pfs47* is polymorphic^30^ and therefore we examined single nucleotide polymorphisms (SNPs) in the ORF and UTRs in sequences of field isolates and laboratory strains available on PlasmoDB (www.PlasmoDB.org) as well as exonic sequences from 3488 isolates from 23 countries on MalariaGen (www.malariagen.net/data/pf3k-4). In HR1 32 SNPs were identified in the 202 laboratory lines and field isolates sequenced and analysed on PlasmoDB. Of these only four SNPs had minor allele frequencies (MAF) above 1%, all others were below 1%. Cloning of the 851 bp HR1 of *pfs47* from 3D7 resulted in six nucleotide deletions in repetitive sequence elements compared to the published sequence, a 99.3% identity level. In HR2 a total of 68 SNPs could be identified with 53 having a MAF of less than 1%, often only found in a single isolate. However, geographic region-specific haplotypes are apparent in lines derived from South America showing six SNPs with fixation or at near fixation frequencies, as well as in parasites originating from South East Asia with six SNPs, three of which are identical to those of the South American isolates (Supplementary Table S1). Of the lines listed in Supplementary Table S1 the highest number of SNPs in any line is 7, a 99.3% level of sequence identity to the HR2. Similarly for *p230p*, we selected homology regions with the minimum number of SNPs (Supplementary Table S2). The first region of homology chosen (500 bp in length) contained 38 SNPs in the Pf3K-4 dataset with 35 of these showing a MAF of 0.1% or less. In the DNA region chosen as HR2 (644 bp in length) 83 SNPs were identified, with only 8 SNPs showing a MAF above 1% and the highest at 7%. To allow modifications in already WR99210-resistant strains we also developed a CRISPR/Cas9 vector containing a *pac*-y*fcu* selection cassette, allowing for positive selection using puromycin (Supplementary Fig. S3).

In order to direct the SpCas9 nuclease to target the *p230p* locus for a DSB, two guide RNA sequences were designed and inserted into the U6 cassette, resulting in two plasmids pDC115 and pDC120. For targeting *pfs47* a single guide RNA was designed and cloned into pDC2-Cas9-hDHFRyFCU, resulting in pDC287. One SNP was identified in the DNA sequence used as guide RNA in pDC115 (C5613T) with a MAF of 0.1% and only detected in lines derived from Bangladesh, whereas the DNA sequence chosen as protospacer in pDC120 showed no SNPs. The protospacer sequence in pDC287 for targeting *pfs47* carries two SNPs (G135C and T148C) with MAFs of 0.4% and 0.5%, respectively.

**Figure 3:**
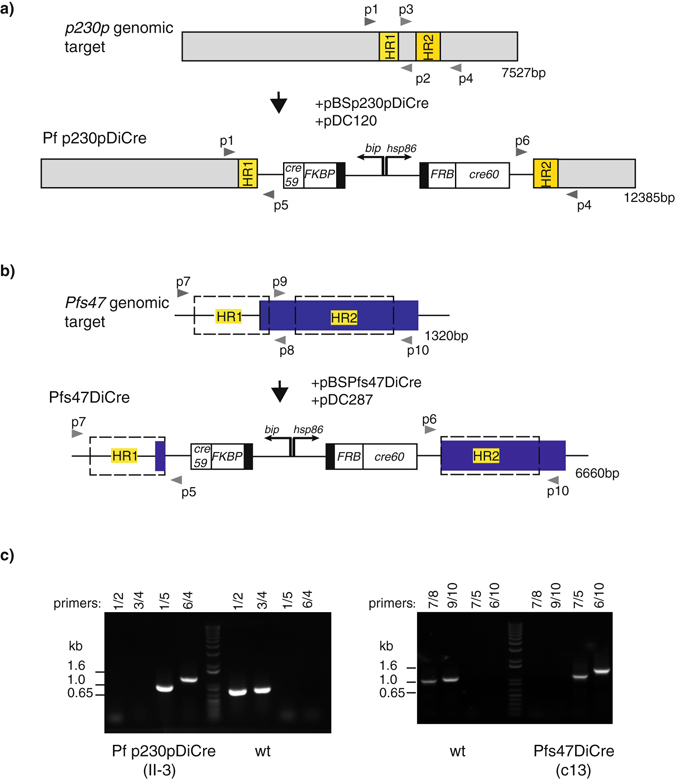
Successful generation of *P. falciparum* parasite lines expressing DiCre recombinase using CRISPR-Cas9. (**a**) Schematic of wild type *p230p* ORF and modified ORF containing the DiCre recombinase expression cassettes following transfection of 3D7 with pBSp230pDiCre and pDC120 (pDC2-hDHFR-yFCU plasmid carrying the guide sequence). Homology regions (HRs) used for targeting were 500 bp (HR1) and 644 bp (HR2) in size. (**b**) Schematic of wild type *pfs47* locus and modified, DiCre-expressing *pfs47* locus. The blue box depicts the *pfs47* ORF, and hashed boxes the DNA regions used for homologous repair. Transfection of 3D7 with pBSPfs47DiCre and pDC287 (the plasmid carrying the guide sequence for *Pfs47* targeting) resulted in the disruption of the *pfs47* ORF. (**c**) PCR screen on wild type and transgenic parasite clones (II-3 and Pfs47-13) showing successful integration of the DiCre cassette into the targeted loci. Primer pairs 1/2 and 3/4 amplify fragments of *p230p* in wild type parasites only with expected sizes of 772 bp and 771 bp respectively. Primer pairs 1/5 and 4/6 amplify DNA only after successful integration into *p230p* with expected sizes of 947 bp and 1229 bp respectively. Primer pairs 7/8 and 9/10 amplify 1042 bp and 1114 bp fragments from the *pfs47* wild type locus only; whereas primer pairs 5/7 and 6/10 only amplify the expected 1257 bp and 1555 bp bands after successful integration. Primers used for amplification are shown on the top of each panel, as well as in the schematics in a) and b). DNA marker sizes are indicated on the left in kb.

Successful transgene integration into *p230p* and *pfs47* will result in the disruption of either genomic locus, as depicted in Fig. 3a and b.

For transfection, we used the Amaxa™ 4D-Nucleofector™ technology with purified 3D7 schizonts, 60 μg of linearised rescue plasmid, and 20 or 30 μg of the CRISPR/Cas9 plasmid carrying the respective guide RNA. Transfection of linear DNA is common practice for generating transgenic *P. berghei* and *P. knowlesi* parasites and has also been used successfully for *P. falciparum* ^5^. This method has the advantage that linear DNA is not retained beyond 4 days post-transfection^31^ and for this reason and the marker-free approach chosen here, hDHFR selection with WR99210 was only applied for 4 days. Viable parasites were detected 14 d post-transfection and subsequently screened for integration by PCR and treated with 5-fluorocytosine (5-FC) for one week prior to cloning by limiting dilution. Eight out of 10 clones of the targeted integration into the *pfs47* locus gave rise to bands of the expected sizes, whereas two out of 26 clones of the targeted integration into the *p230p* locus with either of the two guide RNAs gave rise to the expected PCR products (Fig. 3c). Seven weeks following transfection we had successfully generated marker-free parasite clones with the desired integration of the DiCre-recombinase cassettes into the target genes. Further tests revealed that these clones were sensitive to WR99210, having lost the CRISPR/Cas9 plasmid (data not shown). We selected one clone from each transfection for further analysis: II-3 (*p230p*DiCre) and Pfs47-13 (*pfs47*DiCre).

### *The pfs47*DiCre and *p230p*DiCre *Plasmodium falciparum* lines allow efficient excision of *loxP*-flanked reporter constructs

**Figure 4:**
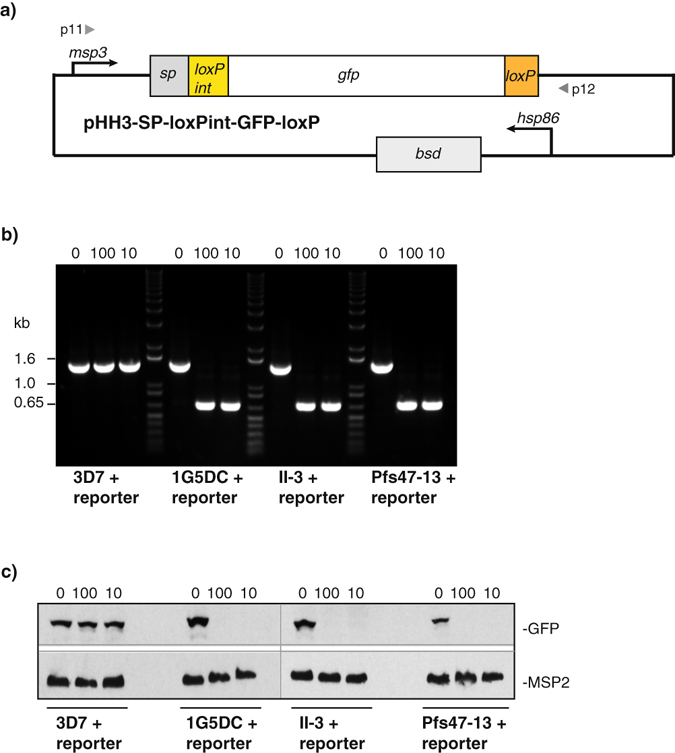
The new transgenic parasite lines excise floxed DNA sequences efficiently. (**a**) Schematic of reporter plasmid pHH3-SP-loxPint-GFP-loxP. The *gfp* ORF is flanked by a *loxPint* site upstream and a *loxP* site downstream. Transcription is driven by 1258 bp of *msp3* 5′UTR sequence. SP, 110 bp of signal peptide-encoding sequence from *msp3*; *bsd*, blasticidin S deaminase driven by *hsp86* promoter. 3D7, and the DiCre-expressing parasite lines 1G5DC, II-3 and Pfs47-13 were transfected with the reporter plasmid and selected using 2.5 µg/ml blasticidin. (**b**) PCR amplification of the GFP cassette using primers 11 and 12 showing successful recombination after rapamycin treatment. The expected product size is 1471 bp before excision and 670 bp following excision. Treatment conditions were either 0.05% DMSO (0), 100 nM rapamycin for 4 h (100) or 10 nM rapamycin for 1 h (10) as indicated on the top of each lane. The parasite lines treated are indicated at the bottom of the panel. DNA marker sizes are shown on the left. (**c**) Immunoblots showing the lack of a 27 kDa GFP band after rapamycin treatment. The four parasite lines transfected with the reporter plasmid were treated with DMSO or rapamycin as indicated on the top of each panel and described in section b). Treatment occurred at early ring stage and schizonts were harvested by Percoll gradient 38–40 h later before separation on SDS-PAGE and immunoblotting. Anti-GFP and anti-MSP2 antibodies were used as indicated on the right of the panels.

**Figure 5:**
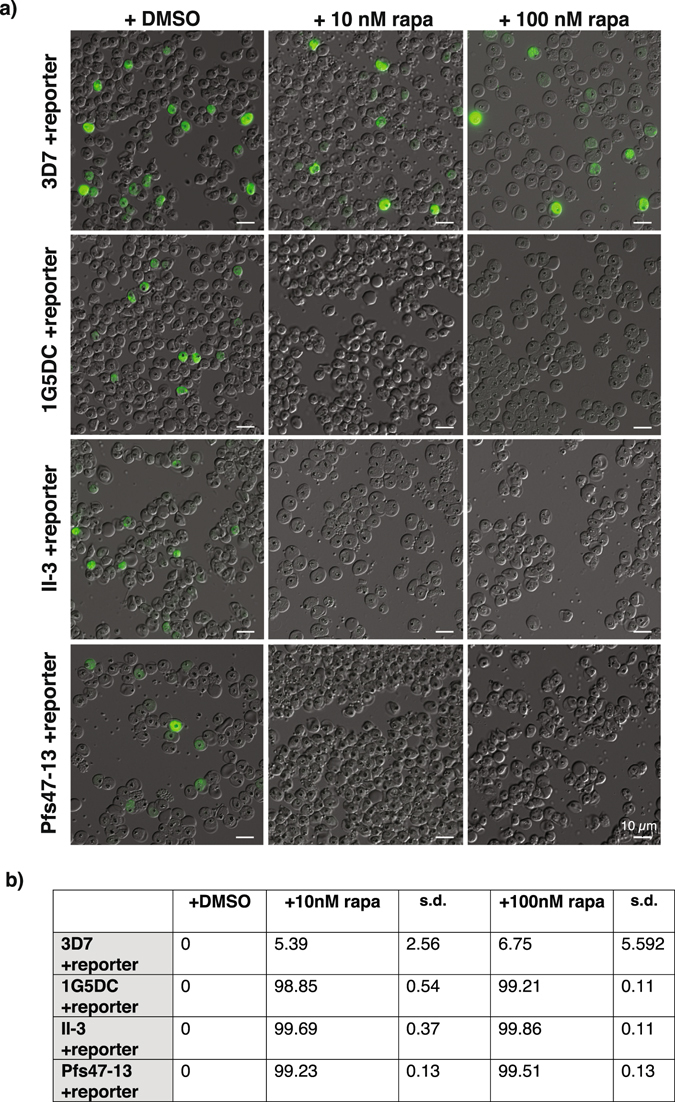
The new transgenic parasite lines expressing DiCre recombinase excise floxed DNA sequences with >99% efficiency using rapamycin at lower concentrations and short treatment times. (**a**) Live GFP fluorescence assay of wild type and DiCre transgenic parasite lines transfected with the reporter plasmid pHH3-SP-loxPint-GFP-loxP and treated at the early ring stage with DMSO or rapamycin as indicated on top of the panels. Images were taken 40 h later. Size bars equal 10 μm. (**b**) Excision efficiency determined by flow cytometry. The four parasite lines transfected with the reporter plasmid were treated with DMSO or rapamycin as described above and the number of GFP positive schizonts was counted 38–40 h after treatment. Data correspond to the mean and standard deviation (s.d.) calculated from three independent experiments counting 100,000 cells each time.

To test whether these new DiCre recombinase transgenic parasites are able to mediate rapamycin-induced excision we transfected wild type 3D7 and the DiCre-expressing parasite lines 1G5DC, II-3 and Pfs47-13 with a GFP reporter plasmid in which *gfp* is flanked by *loxP* sites (Fig. 4a and Supplementary Fig. S4). Treatment of the DiCre-expressing reporter lines with rapamycin induced efficient excision of *gfp* as detected by PCR, immunoblot and fluorescence microscopy (Fig. 4b,c and Fig. 5a), irrespective of rapamycin treatment conditions (100 nM for 4 h or 10 nM for 1 h). Treatment of all reporter lines with DMSO and treatment of 3D7 parasites carrying the reporter plasmid with rapamycin did not induce excision and consequently there was GFP expression.

To quantify the extent of excision we compared numbers of GFP-positive schizonts between DMSO and rapamycin treatments of the same reporter line by flow cytometry. Excision within one replication cycle exceeded 99% in II-3 and Pfs47-13 parasite lines, comparable to the level of excision measured for the established 1G5DC line (Fig. 5b). Equally, rapamycin treatment at lower concentrations and for shorter exposure times resulted in excision levels comparable to those using the standard treatment conditions.

We also investigated the potential for reversion in the DiCre transgenic reporter lines by PCR (Supplementary Fig. S5). II-3 and Pfs47-13 DiCre parasite lines generated by double homologous recombination following CRISPR/Cas9 induced DSBs showed no reversion at the *p230p* or *pfs47* locus, respectively. On the other hand after three months in culture, PCR amplification from the 1G5DC reporter line showed a band corresponding to an intact *SERA5* locus, suggesting that over time the DiCre cassette is lost in part of the population. These results show that the new transgenic *P. falciparum* lines II-3 and Pfs47-13 stably maintain DiCre recombinase and excise *loxP*-flanked DNA sequences with extremely high efficiency even at a 10-fold lower rapamycin concentration than has been routinely used.

## Discussion

The aim of this study was to develop a complete toolkit to generate rapamycin-inducible DiCre recombinase-expressing *P. falciparum* lines which efficiently and rapidly excise floxed DNA sequences in any genetic background.

CRISPR/Cas9 gene editing tools in *Plasmodium* spp. were first reported in 2014^5–7^. Since then further improvements to the system have been made, allowing for quick, marker-free generation of transgenic parasites. These techniques have proven a powerful tool, shortening the time from transfection to analysis of transgenic parasite clones by many months. Multiple successive rounds of gene modification in the same parasite are therefore now realistically achievable. In this study we have further improved the CRISPR/Cas9 system and transfection methods using linear DNA for marker-free modifications in *P. falciparum*, resulting typically in the generation of transgenic parasites and the isolation of clones lacking integrated drug selection cassettes in less than two months, the fastest method so far reported for *P*. *falciparum*. Our approach is similar to that of both Lu *et al*.^29^ and Mogollon *et al*.^24^. We have used two plasmids, a CRISPR/Cas9 plasmid encoding the single guide RNA and SpCas9 nuclease, and a rescue plasmid, carrying the regions for repair by homologous recombination. *Plasmodium* species, like a few other eukaryotic pathogens (*Giardia lamblia, Trichomonas vaginalis* and *Trypanosoma brucei*), lack efficient NHEJ as a DNA repair mechanism (reviewed in Lee *et al*.^32^), instead using almost exclusively homologous recombination^26, 33^. Our CRISPR/Cas9 plasmid, pDC2-Cas9-hDHFRyFCU, carries a positive and a negative selection cassette in the form of a h*dhfr*-y*fcu* fusion gene, whereas the rescue plasmid is derived from pBluescript and carries no drug selection, allowing for the insertion of large DNA fragments. This system has the advantage that no selection marker is integrated into the chromosome and addition of 5-FC to cultures allows for the selection of parasites that have lost the CRISPR/Cas9 vector. In addition, we linearised the rescue plasmid for transfection to avoid potential maintenance as an episome even though it carries no drug resistance marker. To maximise the uptake of both plasmids into the same parasite we transfected a molar ratio of 2.5:1 to 4:1 of rescue to CRISPR/Cas9 plasmids and only applied selection to the cultures for 4 days using WR99210. The use of hDHFR as a positive selection marker over blasticidin S deaminase (*bsd*) is preferable as no development of spontaneous resistance to WR99210 has been reported, whereas the use of blasticidin S can result in the selection for spontaneous blasticidin S resistance in *P. falciparum* cultures^34, 35^.

Using this improved CRISPR/Cas9 system we generated functional DiCre recombinase-expressing 3D7 *P. falciparum* parasites by inserting the DiCre cassettes into the genomic locus of *p230p* and *pfs47*, yielding parasite lines called II-3 (*p230p*DiCre) and Pfs47-13 (*pfs47*DiCre), respectively. Using double homologous recombination for integration of the cassettes circumvents the possibility of spontaneous loss of the DiCre recombinase cassettes as seen in the 1G5DC line. Furthermore, the *p230p* and *pfs47* loci were chosen as disruption of these genes does not alter asexual blood stage growth and development or gametocyte production^24, 25^. In fact, *p230p* in rodent malaria parasites also seems dispensable for development in the mosquito stages^36^. *P. falciparum pfs47* knockout parasites have been described as developing in *Anopheles stephensi* but seem unable to establish successful infections in *A. gambiae* ^36, 37^.

All DNA sequences chosen as homology regions contained SNPs, mostly with low MAFs. In laboratory-adapted lines the sequence disparity was less than 1% and we expect no difficulties in generating DiCre recombinase-expressing parasites in these using the plasmids described here^26^. Given the low MAFs of SNPs we equally expect these plasmids to be useful for generating DiCre-expressing parasite lines from field isolates. In fact, in the plasmid pBSPfs47DiCre the homology region one lacks six nucleotides compared to the published 3D7 sequence, a 99.3% identity level, and we achieved transgene integration in the *pfs47* locus in 80% of the population after transfection. Targeting the *p230p* locus with either of two different guide RNAs was less efficient and resulted in only 8% of the clones carrying the DiCre cassettes. We attribute this difference in efficiency of gene editing to the nature of the guide RNAs selected. We have used pDC-Cas9-hDHFRyFCU to modify numerous *P. falciparum* genes and found differences in efficiency in modifying a locus are dependent on the guide RNA sequences selected, a principle generally applicable for CRISPR/Cas9 gene editing^38^. We succeeded in our first attempt to integrate the transgenes into both the *pfs47* and the *p230p* locus. Independent transfections targeting the *pfs*47 locus by us and others resulted in 86% (n = 1) and 50 to 58% (n = 4) of clones expressing DiCre (Tiburcio and Treeck, personal communication). Together these data indicate that the plasmids in this toolkit allow for the efficient production of the desired DiCre recombinase-expressing parasite lines with minimal effort. When modifying field isolates, however, we suggest prior examination of the *p230p* or *pfs47* DNA sequences, especially for guide RNA design to avoid possible SNPs, however rare.

The DiCre cassettes used in this study are based on those reported by Collins *et al*.^13^ that use the *hsp86* and *bip* promoters to drive transcription of the split *cre* genes. Studies showing lower excision levels of 50 to 80% have maintained the DiCre-recombinase expression cassettes on episomes and used the *hsp86* and *ef1α* promoters to drive transcription of the split *cre* genes. Although these promoters allow for constitutive transcription, the *ef1α* promoter is very weak compared to the *hsp86* promoter, which probably does not allow sufficient DiCre recombinase expression early in the life cycle. Coordinated expression of both parts of the DiCre recombinase early in the life cycle appears to be crucial for extensive *loxP*-guided recombination^12^. This difference might explain the superior excision levels seen using the DiCre cassettes reported by Collins *et al*.^13^ and used here to generate our transgenic parasites II-3 and Pfs47-13.

The DiCre-recombinase system as a conditional gene regulation system is becoming more widely used. Tamoxifen-induced dimerisation of estrogen receptor binding domains fused to split Cre as well as photoactivatable dimerisation have been used to bring both halves of the split Cre recombinase together to form an active recombinase in mice^39, 40^. The system established for *P. falciparum* uses the rapamycin-binding FKBP12 and FRB proteins to dimerise the two enzyme halves. Rapamycin and also the carrier DMSO are toxic to *P. falciparum* at higher concentrations^14, 41^. For that reason we have established the lowest concentration of rapamycin and the shortest exposure time required to achieve maximal recombination levels: 10 nM rapamycin for 30 min. This protocol results in excision levels still exceeding 99% with no impact on parasite growth and is cheaper and more user-friendly compared to the standard treatment of 100 nM rapamycin for 4 h. We also wanted to understand whether the timing of excision after addition of rapamycin at early ring stages is affected by the concentration of rapamycin and exposure period. We detected no difference whether using a 10-fold higher rapamycin concentration for a longer period or the minimal treatment conditions we established here. In both cases it took about 20 h before significant levels of excision could be detected and more than 30 h to achieve maximum levels. A delay between rapamycin exposure and start of excision of floxed DNA sequences has been reported previously^13, 14, 17^. This delay could reduce the usefulness of this conditional regulation system for genes transcribed in the first 24 h of the life cycle. An example of this has been reported recently using conditional DiCre recombinase-mediated excision of *kelch13* and *rab5a* ^12^. After addition of rapamycin no Rab5a could be detected later in that cycle whereas Kelch13 persisted for at least two days, although at much reduced level. *Kelch13* is transcribed in schizont and ring stages, whereas *rab5a* mRNA level seems to peak in trophozoite stages. Therefore translation of *kelch13* was on going during DiCre recombinase induction, and for stable proteins with long half-life this can result in masking of the phenotype for one or more cycles. To circumvent this additional treatment with rapamycin later in the life cycle or prolonged exposure to rapamycin might be explored. Other regulation systems however based on RNA or protein stability might be more suitable in these circumstances even though they are more variable in the regulation achieved. Alternatively, protein mislocalisation by knock-sideways approaches might be employed to study the function of proteins translated early in the life cycle. However, this system will not work for secreted proteins or those rendered dysfunctional by addition of multiple FKBP-tags at the N- or C-terminus^12^. For the study of gene products expressed during trophozoite and/or schizont stages of the parasite and their function, the DiCre system is able to achieve an unparalleled level of regulation with near 100% loss of the gene in one replication cycle, making it the system of choice.

We believe the toolkit of plasmids presented here will lead to the speedy generation of DiCre-expressing transgenic parasites allowing for the rapid and efficient rapamycin-inducible excision of genes and the study of their function during one growth cycle. We envisage the toolbox will be used to generate DiCre recombinase-expressing parasites in strains showing drug resistance, in strains with defined red cell invasion pathways, recently adapted field isolates and in strains with the potential to undergo gametocytogenesis and transmission to mosquitoes, thereby opening the possibility of studying gene product function in parasites during the mosquito life cycle in an inducible manner.

## Methods

### Parasite culture and transfection

*P. falciparum* parasites were grown *in vitro* in RPMI 1640 medium containing 0.5% w/v AlbumaxII as described^42^. Parasites were synchronised by purifying schizont stages using a 70% Percoll gradient, before allowing reinvasion to occur, and followed by sorbitol treatment. All parasites used in this study were derived from the 3D7 clone, including the control 1G5DC strain^13^. Plasmids were introduced into purified schizont stage parasites by electroporation using an Amaxa 4D-Nucleofector™ (Lonza) as previously described^43^. For transfections with reporter plasmids to be maintained episomally 10 μg of DNA was electroporated and cultures were selected continuously with 2.5 μg/ml of blasticidin-S-HCl. For generation of marker-free, DiCre transgenic parasites 20–30 μg of CRISPR/Cas9 plasmids and 60 μg of linearised rescue plasmids were electroporated. Selection was applied one day after transfection with 5 nM WR99210 (a kind gift of Jacobus Pharmaceuticals) for four days. Following establishment of transgenic parasites, the cultures were treated with 1 μM 5-fluorocytosine (5-FC) provided as clinical grade Ancotil^®^ (MEDA) for one week before parasite cloning by limiting dilution. To induce DiCre recombinase mediated excision of DNA, early ring stage parasites were treated with rapamycin (stock solution was 200 μM in DMSO), before being washed in warm RPMI 1640 medium and returned to culture.

### Cloning of CRISPR/Cas9 plasmids and insertion of guide RNA sequences

The pDC2-Cas9-U6-h*dhfr* vector^27^ (GenBank accession number KY574493) was modified by replacing the h*dhfr* with h*dhfr-*y*fcu*. h*dhfr*-y*fcu* was amplified from pL0034^28^ as three overlapping PCR products using primer pairs DHFRyFCUfor/Y1rev, Y1for/Y2rev and Y2for/DHFRyFCUrev. PCR products were purified and used at an equimolar ratio as template in the subsequent PCR reaction with primer pair DHFRyFCUfor/DHFRyFCUrev. The 1746 bp product was gel-extracted and cloned into pDC2-Cas9-U6-h*dhfr* vector using NcoI/SacII sites resulting in plasmid pDC2-Cas9-hDHFRyFCU. For generating a CRISPR/Cas9 vector with a different positive selection we introduced into pDC2-Cas9-U6-h*dhfr* a *pac-*y*fcu* cassette replacing the h*dhfr* cassette. The gene for puromycin N-acetyltransferase (*pac*) was amplified from mPAC-TK (kind gift of Alex Maier) using primer pair PACforNcoI/PACrev(int). The y*fcu* gene was amplified from pDC2-Cas9-hDHFRyFCU using primer pair Linkerintfor/DHFRyFCUrev. The *pac*-y*fcu* fusion gene was finally generated by overlapping extension PCR and cloned into pDC2-Cas9-U6-h*dhfr* using NcoI/SacII restriction sites.

Potential guide RNA sequences were identified using Protospacer software (www.protospacer.com, versions 0.0.1 alpha and 0.1.0 beta^44^). A pair of complementary oligonucleotides corresponding to the 19 nucleotides closest to the identified PAM sequence was synthesized, phosphorylated using T4 polynucleotide kinase, annealed and ligated into pDC-Cas9-hDHFRyFCU digested with BbsI. To generate compatible, sticky ends between the annealed primer pair encoding the guide RNA and the BbsI digested vector the forward oligonucleotide had 5′-ATTG added to the 19 nucleotides corresponding to the guide RNA whereas the compatible oligonucleotide had a 5′-AAAC overhang added (see supplementary Table S3). In this way guide RNAs for targeting *p230p* were assembled using oligonucleotide pairs 115for/rev and 120for/rev and cloned into pDC-Cas9-hDHFRyFCU resulting in CRISPR/Cas9 plasmids pDC115 and pDC120 respectively. In order to target the *pfs47* locus guide RNA oligonucleotide pair 287for/rev was phosphorylated, annealed and ligated into pDC-Cas9-hDHFRyFCU resulting in vector pDC287.

### Construction of rescue plasmids carrying a DiCre recombinase expression cassette

The plasmid carrying the ORFs for the Cre59 and Cre60 domains of the DiCre recombinase fused to nuclear targeting sequences^17^ as well as all the *P. falciparum*-specific 5′ and 3′-regulatory elements is based on pBS_DC_hsp86/BiP5′^13^. Homology regions for *p230p* were amplified from 3D7 genomic DNA using primer pairs p230pF5for/p230pF5rev and p230pF6for/ p230pF6rev and inserted into pBS_DC_hsp86/BiP5′ using restriction sites SacI/AflII and SpeI/KpnI, respectively, yielding plasmid pBSp230pDiCre. Homology regions of the *pfs47* locus were amplified using primer pairs CVO115/CVO116 and CVO117/CVO118 and inserted using restriction sites SacI and SpeI/KpnI, respectively, resulting in pBLD466 (synonymously named pBSPfs47DiCre). For transfection of 3D7 schizonts 60 μg of pBSp230pDiCre and pBSPfs47DiCre were linearised with ScaI for 3 h at 37 °C before heat inactivation at 80 °C for 20 min. 20–30 μg of single guide RNA plasmids were added and the plasmid mixture ethanol precipitated, washed and resuspended in 10 μl sterile 10 mM Tris, 1 mM EDTA (TE) buffer.

### Cloning of reporter plasmid pHH3-SP-loxPint-GFP-loxP

pHH3-SP-loxPint-GFP-loxP reporter plasmid is based on vector pHH3bsdMSP3promGFP^45, 46^ which was digested with AvrII/SacII to release GFP. A DNA cassette encoding the MSP3 signal peptide - loxPint - *gfp* ORF sequence –*loxP* was amplified with primer pair MSP3SPfor/GFPloxPrev from transgenic A3-5 parasites after rapamycin treatment. The A3-5 strain is an inducible Δ*msp3*Δ*msp6* parasite strain in which both *msp* genes are replaced with *gfp* after DiCre-mediated excision, only maintaining the first 110 bp of the *msp3* ORF which encode the signal peptide sequence (Knuepfer and Holder, unpublished). The PCR product was purified, digested with NheI/SacII and ligated into pHH3bsdMSP3prom resulting in the desired reporter plasmid. All constructs were verified by DNA sequencing.

### Nucleic acid extraction and polymerase chain reaction

For DNA extraction total cell pellets were first treated with 0.15% saponin in PBS for 10 min on ice, then washed in PBS before DNA was extracted using a QIAamp DNA Blood Mini kit (Qiagen). For diagnostic PCR amplification we used GoTaq® (Promega) DNA master mix; for amplification of fragments used in construct design we used Phusion® high fidelity DNA polymerase (NEB).

### SDS-PAGE and immunoblotting

Parasite lines were treated with rapamycin or DMSO at early ring stage and schizonts were purified by Percoll gradient ~40 h later. Schizont pellets were lysed in SDS sample buffer containing 100 mM DTT before protein separation on precast Bis Tris NuPAGE polyacrylamide gels and transferred to nitrocellulose membranes by electroblotting. Blots were blocked overnight and incubated first with anti-GFP antibody (Roche) 1:1000 followed by horseradish peroxidase-conjugated secondary antibody (Biorad). Bands were detected using ECL Plus Western blotting reagent (GE Healthcare) and Kodak BioMax MR film. The same blots were subsequently incubated with anti-MSP2 antibody mAb113.1^47^.

### Fluorescence and FACS analysis

Parasite cultures were treated with rapamycin or 0.05% DMSO at early ring stage; schizonts were purified 40 h later using a Percoll gradient. Live, purified schizonts carrying the reporter plasmid and expressing GFP were visualised using a Nikon Eclipse Ni microscope with LED-illumination and a 63x Plan Apo λ NA 1.4 objective. Images were taken using an Orca Flash 4 digital camera controlled by Nikon NIS Elements AR 4.30.02 software. To quantitate excision rates by flow cytometry GFP reporter-expressing parasite lines were treated with rapamycin or DMSO, and purified 40 h later as described above before being stained with Hoechst 33342 at 5 μg/ml for 10 min. Stain was removed and percent GFP-positive schizonts was determined in three separate experiments using a BD FACSAria™ Fusion and BD FACSDiva software v8.0.1. Percentage excision rate was determined as follows: 100 − (% GFP positive after rapamycin treatment/% GFP positive after mock treatment) × 100. All reporter lines were maintained using 2.5 μg/ml blasticidin-S-HCl throughout.

### SNP analysis of DNA regions used for homologous recombination

Open reading frame and 5′UTR DNA sequences of *pfs47* and *p230p* were obtained from PlasmoDB (www.plasmodb.org) and aligned using ClustalW in BioEdit (www.mbio.ncsu.edu/bioedit/bioedit.html). Of the sequences for 10 laboratory lines available we chose 8, excluding V1_S due to large sequence gaps and the IT line due to multiple frame shifts in *pfs47* ORF. We chose further to analyse, align and display 19 sequences obtained from field isolates from French Guiana as both ORF and 5′UTR sequences were complete without sequencing gaps. In addition, we analysed SNPs found in HRs from ORF sequences in data available from the Pf3K Project (2015): pilot data release 4 (www.Malariagen.net/data/pf3k-4). Polymorphisms in the 5′UTR region of *pfs47* used in HR1 in pBSPfs47DiCre were analysed using a total of 202 laboratory lines and field isolate sequences available on PlasmodDB.

### Data availability statement

All plasmids and transgenic *P. falciparum* parasites described in this study are available upon request.

## Electronic supplementary material

Supplementary Information
