# Supplementary material for: Generating conditional gene knockouts in Plasmodium – a toolkit to produce stable DiCre recombinase-expressing parasite lines using CRISPR/Cas9
Source: Sci Rep. 2017 Jun 20;7:3881. doi: 10.1038/s41598-017-03984-3 (PMC5478596; doi:10.1038/s41598-017-03984-3)

Generating conditional gene knockouts in *Plasmodium* – a toolkit to produce stable DiCre recombinase-expressing parasite lines using CRISPR/Cas9

Ellen Knuepfer[1*], Marta Napiorkowska[2†], Christiaan van Ooij[2*], Anthony A. Holder[1*]

Malaria Parasitology[1] and Malaria Biochemistry[2] Laboratories, The Francis Crick Institute, 1 Midland Road, London, NW1 1AT, United Kingdom

† Current address: Department of Biosystems Science and Engineering, ETH Zurich, Mattenstrasse 26, Basel 4058, Switzerland

Supplementary Table 1. Polymorphism in *Pfs47* gene

DNA sequences from *P. falciparum* lab strains and 19 field isolates from French Guiana (obtained from PlasmoDB.org) from diverse geographical regions were analysed for SNPs within the *pfs47* ORF and the 5'UTR region. Nucleotide positions are listed on the top of the table based on ORF numbering in the 3D7 reference sequence; HR2 is the second homology region. Dots indicate identical nucleotides based on the 3D7 reference sequence. The SNPs at positions 532, 581, 707, 725, 740 and 742 are at fixation or near fixation frequencies in lines from South America.

Supplementary Table 2. Polymorphism in *p230p* gene

DNA sequences from *P. falciparum* lab strains and 19 field isolates from French Guiana (obtained from PlasmoDB.org) from diverse geographical regions were analysed for SNPs within the *p230p* ORF region 4979 – 6744 bp. Nucleotide positions are listed on the top of the table based on ORF numbering in the 3D7 reference sequence; HR2 is the second homology region. Dots indicate identical nucleotides based on the 3D7 reference sequence.

Supplementary Table 3. Primer list

List of the oligonucleotides used in this study and their DNA sequences. Restriction enzyme recognition sites are underlined. Four base pair additions to create sticky ends and allow ligation of guide RNA pairs into pDC-Cas9-hDHRFyFCU vector are shown in bold. The *loxP* sequence is shown in italics.

Supplementary Figure S1. Plasmid map of pDC2-Cas9-hDHFRyFCU

This vector is derived from pDC2-Cas9-U6-h*dhfr* [1] (GenBank accession number KY574493) and encodes the Cas9 nuclease as well as the single guide RNA. Cas9 is driven by the calmodulin promoter; the positive (h*dhfr*) and negative (y*fcu*) selection cassette are fused and driven by a single, constitutively active promoter (*PcDT*, *P. chabaudi* dihydrofolate reductase-thymidylate synthase). A 549 bp *U6* promoter drives the transcription of the protospacer-tracrRNA fusion (sgRNA) and is followed by a 52 bp *U6* terminator. Protospacers were cloned into the guideRNA cassette using BbsI sites.

Supplementary Figure S2. Maps for rescue plasmids pBSp230pDiCre and pBSPfs47DiCre.

Regions of DNA sequence homology to *p230p* and *pfs47* are indicated in yellow. The DiCre expression cassette is unaltered from pBS_DC_hsp86/BiP5' [2] with the Cre60 domain driven by the *hsp86* promoter and the Cre59 domain by the *bip* promoter. Nuclear localisation sequences are indicated in dark blue.

Supplementary Figure S3. Plasmid map of pDC2-Cas9-PACyFCU

This CRISPR/Cas9 vector encodes the Cas9 nuclease and the single guide RNA cassette. This vector is a derivative from pDC2-Cas9-U6-h*dhfr* and contains the positive drug selection cassette puromycin N-acetyltransferase (*pac*) and the negative selection (y*fcu*)

Supplementary Figure S4. Plasmid map for reporter plasmid, pHH3-SP-loxPint-GFP-loxP

The open reading frame of *gfp* is indicated in green and flanked by two *loxP* sites (yellow), of which the upstream site is located within the *SERA2* intron (*loxPint*). The brown box represents 110 bp of the *msp3* ORF encoding the predicted signal peptide sequence and cleavage site. The reporter cassette is driven by 1258 bp of the *msp3* promoter for transcription during schizogony.

Supplementary Figure S5. DiCre recombinase disrupted *p230p* and *pfs47* loci show no reversion to wild type

PCR amplification of the transgene-disrupted loci *p230p*, *pfs47* and *SERA5.* Genomic DNA was isolated from rapamycin-treated reporter lines 3D7,

1G5DC, Pfs47-13 and II-3 transfected with pHH3-SP-loxPint-GFP-loxP maintained on 2.5 μg/ml blasticidin which have been in continuous culture for 3 months. To detect the restored endogenous locus as a result of reversion and loss of the DiCre cassette in a subset of the parasite population, PCR was performed with the following primer pairs: 9/10 amplifying a 1114 bp region of *pfs47* ORF, 1/2 amplifying a 772 bp region of *p230p* ORF and SERA5reversion for/rev amplifying a 1119 bp region of *sera5*. DNA size markers are indicated on the left.

References:

1. Lim, M. Y.-X. *et al.* UDP-galactose and acetyl-CoA transporters as *Plasmodium* multidrug resistance genes. *Nature Microbiology* **1,** 16166 (2016).
2. Collins, C. R. *et al.* Robust inducible Cre recombinase activity in the human malaria parasite *Plasmodium falciparum* enables efficient gene deletion within a single asexual erythrocytic growth cycle. *Mol Microbiol* **88,** 687–701 (2013).

Supplementary Table S1:

					HR2										
			**82**	**163**	**203**	**532**	**581**	**657**	**671**	**707**	**718**	**725**	**740**	**742**	**814**
Africa	Laboratory lines	3D7	**C**	**G**	**C**	**A**	**C**	**G**	**T**	**C**	**C**	**C**	**T**	**A**	**A**
		GB4	.	.	.	.	A	.	.	.	.	.	.	.	.
Latin America	Field Isolates from French Guiana	G224	.	.	T	G	A	.	.	T	.	T	C	T	.
		H209	.	.	.	.	A	.	.	T	.	T	C	T	.
		N011-A	.	.	.	.	A	.	.	T	.	T	C	T	.
		N023-A	.	.	T	G	A	.	.	T	.	T	C	T	.
		N071-I	.	.	.	.	A	.	.	T	.	T	C	T	.
		N164-A	.	.	.	.	A	.	.	T	.	T	C	T	.
		N497-C	.	.	.	.	A	.	.	T	.	T	C	T	.
		N579-A	.	.	.	.	A	.	.	T	.	T	C	T	.
		O079-B	.	.	.	.	A	.	.	T	.	T	C	T	.
		O141-A	.	.	.	.	A	.	.	T	.	T	C	T	.
		O222-A	.	.	T	G	A	.	.	T	.	T	C	T	.
		O306-A	.	.	.	.	A	.	.	T	.	T	C	T	.
		O314	.	.	.	.	A	.	.	T	.	T	C	T	.
		P164-C	.	.	.	.	A	.	.	T	.	T	C	T	.
		P167-B	.	.	T	G	A	.	.	T	.	T	C	T	.
		P237-C	.	.	.	.	A	.	.	T	.	T	C	T	.
		P241-D	.	.	T	G	A	.	.	T	.	T	C	T	.
		PS103	.	.	.	.	A	.	.	.	.	.	.	T	T
		PS122_G11	.	.	.	.	A	A	.	.	.	.	.	.	.
	Laboratory lines	7G8	.	.	.	.	A	.	.	T	.	T	C	T	.
		CS2	A	A	.	.	A	.	A	T	A	.	.	T	T
		SantaLucia	.	.	.	G	A	.	.	T	.	T	C	T	.
		HB3	.	.	.	G	A	.	.	T	.	T	C	T	.
South-East Asia		Dd2	A	A	.	.	A	.	A	T	A	.	.	T	T
		T9_94	A	A	.	.	A	.	.	T	A	.	.	T	T

Supplementary Table S2:

				HR2		
			5944	6264	6432	6531
Africa	Laboratory lines	3D7	**G**	**G**	**T**	**G**
		GB4	A	.	.	T
Latin America	Field Isolates from French Guiana	G224	.	A	.	.
		H209	.	A	.	.
		N011-A	.	A	.	.
		N023-A	.	A	.	.
		N071-I	.	A	.	.
		N164-A	.	A	.	.
		N497-C	.	A	.	.
		N579-A	.	A	.	.
		O079-B	.	A	.	.
		O141-A	A	.	C	.
		O222-A	.	A	.	.
		O306-A	.	A	.	.
		O314	.	A	.	.
		P164-C	.	A	.	.
		P167-B	A	.	C	.
		P237-C	.	A	.	.
		P241-D	.	A	.	.
		PS103	.	.	C	.
		PS122_G11	A	.	.	.
	Laboratory lines	7G8	A	.	.	.
		CS2	A	.	.	.
		SantaLucia	A	.	.	.
		HB3	A	.	.	.
South-East Asia		Dd2	A	.	.	.
		T9_94	A	.	.	.

Supplementary Table S3:

oligo name	alternative name	DNA sequence 5' - 3'	restriction enzyme sites (underlined)
p1	FL5extfor	CCATATCTTTTGATACTTCGGTAAATGTAC	
p2	FL5middle rev	ATGGTTTATCATATGTACATCATGACCTTG	
p3	FL6middle for	ATGTGCTAGGCATATCTACAATGTCTC	
p4	FL6ext rev	TTGCTCATATTCTGATGTGGTAGTATCTG	
p5	Cam3'utrseq2	CCATTTACACATAAATGTCACACAAAAGAG	
p6	Hsp865'utr seq1	GAAATAATTTCATATACACACATATAC	
p7	Pfs47extfor	ATTGCATACACATAAATATTTGTGTTGTAC	
p8	Pfs47intrev	CACATACGTATTGTGTTGAGCTTAATAG	
p9	Pfs47intFor	CTAATGTTAAGCCAACTGTAGTTGGG	
p10	Pfs47extrev	GATGCGATATGTAATTCCATTACTGC	
p11	MSP3promseq2	ATTTGCACACATTATTACGTCTAAATC	
p12	Hrp2seq3	GATTCATTATTCTATATTTATAAGGAAGATTAC	
p13	CVO001	GGACCTCGAGATGAGTTTAAAAAGAAGAAAATTTATTTTGTTGTC	XhoI
p14	CVO097	CATTTAAACCTTTCCAATATCACACATTTGC	
115for	P230pguide115Leefor	**ATTG**TCGAAATTGAACTTATTGA	
115rev	P230pguide115Leerev	**AAAC**TCAATAAGTTCAATTTCGA	
120for	P230pguide120Leefor	**ATTG**AAAATGATATTGTCGGATT	
120rev	P230pguide120Leerev	**AAAC**AATCCGACAATATCATTTT	
287for	pDC287for	**ATTG**ATGTTAAGCCAACTGTAGT	
287rev	pDC287rev	**AAAC**ACTACAGTTGGCTTAACAT	
DHFRyFCUfor		CCTTAACCATGGTTGGTTCGCTAAACTGCATCG	NcoI
DHFRyFCUrev		TTACTTCCGCGGTTAAACACAGTAGTATCTGTCACC	SacII
Y1for		GATTATGGGTAAGAAAACCTGGTTCTCCATTCCTGAG	
Y1rev		CTCAGGAATGGAGAACCAGGTTTTCTTACCCATAATC	
Y2for		CCAGAGGATATATCTGAAAGGTATGTATTCCTATTAGACCCA	
Y2rev		TGGGTCTAATAGGAATACATACCTTTCAGATATATCCTCTGG	
MSP3SPfor		AACCTAGCTAGCATGAAAAGTTTTATAAATATTACTCTTTCATTATTTTTG	NheI
GFPloxPrev		AATATTCCGCGG*ATAACTTCGTATAATGTATGCTATACGAAGTTAT*TTATTTGTATAGTTCATCCATGCCATGTGTAATCCC	SacII
P230pF5for		AATAAGAGCTCATGAATATAAAGAAAAAGAAACTTCGGATAAAATCAAACG	SacI
P230pF5rev		AATAACTTAAGTAACGTTGAAGCTGACGATAATGTTGATGG	AflII
P230pF6for		AATGGACTAGTACAAAAAAGTATCAAGAAAAAAAAATCACAAGGAGG	SpeI
P230pF6rev		AATAAGGTACCGTGTACAGAAGAACGACTAGATGATATATTATCTCC	KpnI
CVO115		GGACGAGCTCCGTTTAAGTACATATTTGTATATGCACACATAAATTTTTCC	SacI
CVO116		GGACGAGCTCGCTGATCATTCTTCCCATACAC	SacI
CVO117		GGACACTAGTGTACTATAAACAACCCTACGTTGGGTGACC	SpeI
CVO118		GGACGGTACCGGTAAGGCTAACATATACATGCCTTCC	KpnI
SERA5reversion for		CAAATGGTACAACTGGTGAACAAGG	
SERA5reversion rev		CTAAGGTACCTGTTGTATCTACGTC	
PACfor NcoI		CCTTAACCATGGATGACTGAATACAAACCAACTGTTC	NcoI
PACrev(int)		TCCACCAGATCCACCTCCTCCGCTAGCAGCACCTGGTTTGCGAGTCATGC	
LinkerintFor		GCTAGCGGAGGAGGTGGATCTGGTGGAGGTGG	

Supplementary Figure S1:

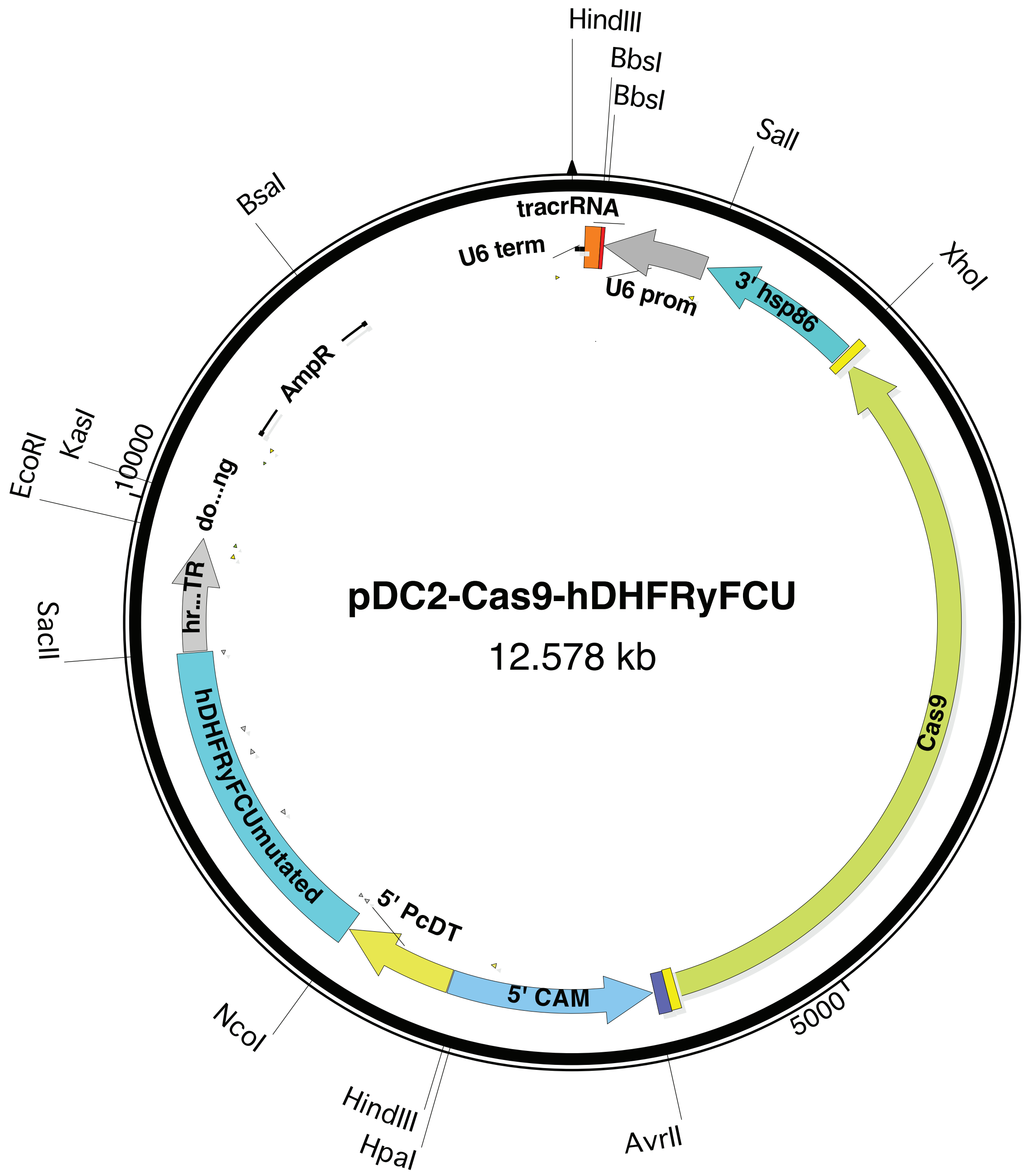

Supplementary Figure S2:

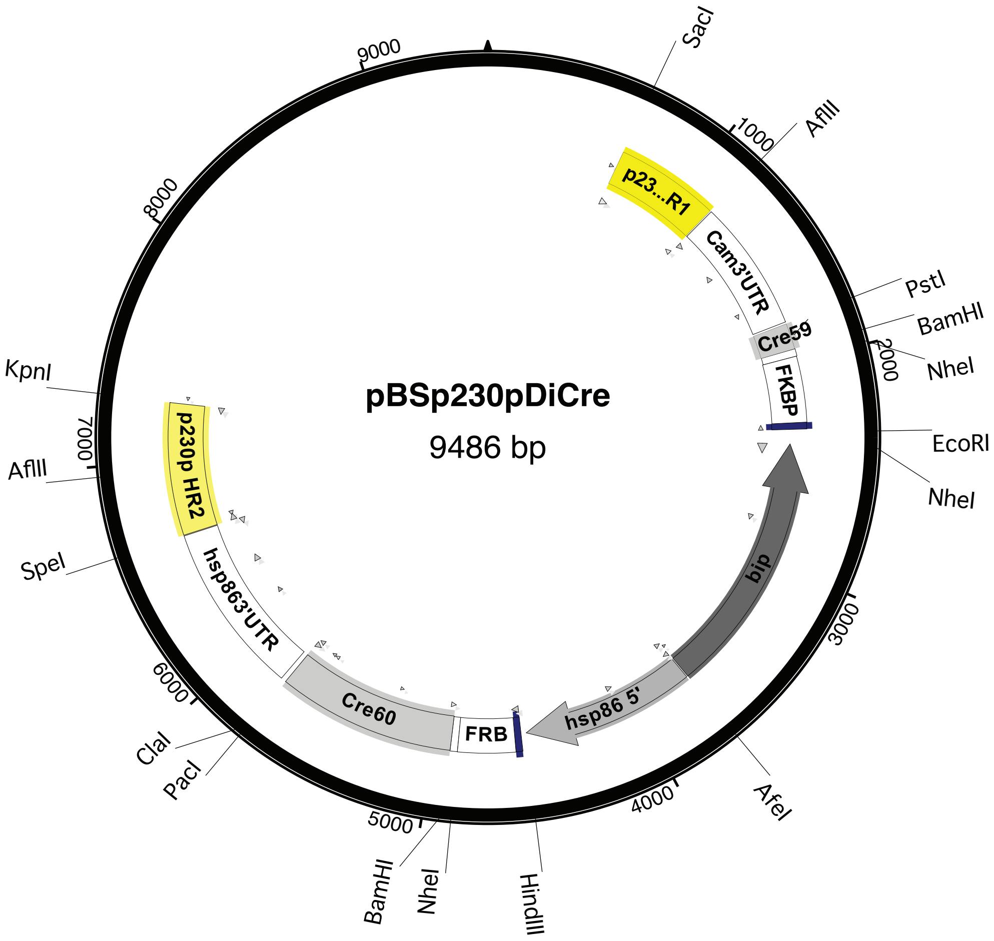

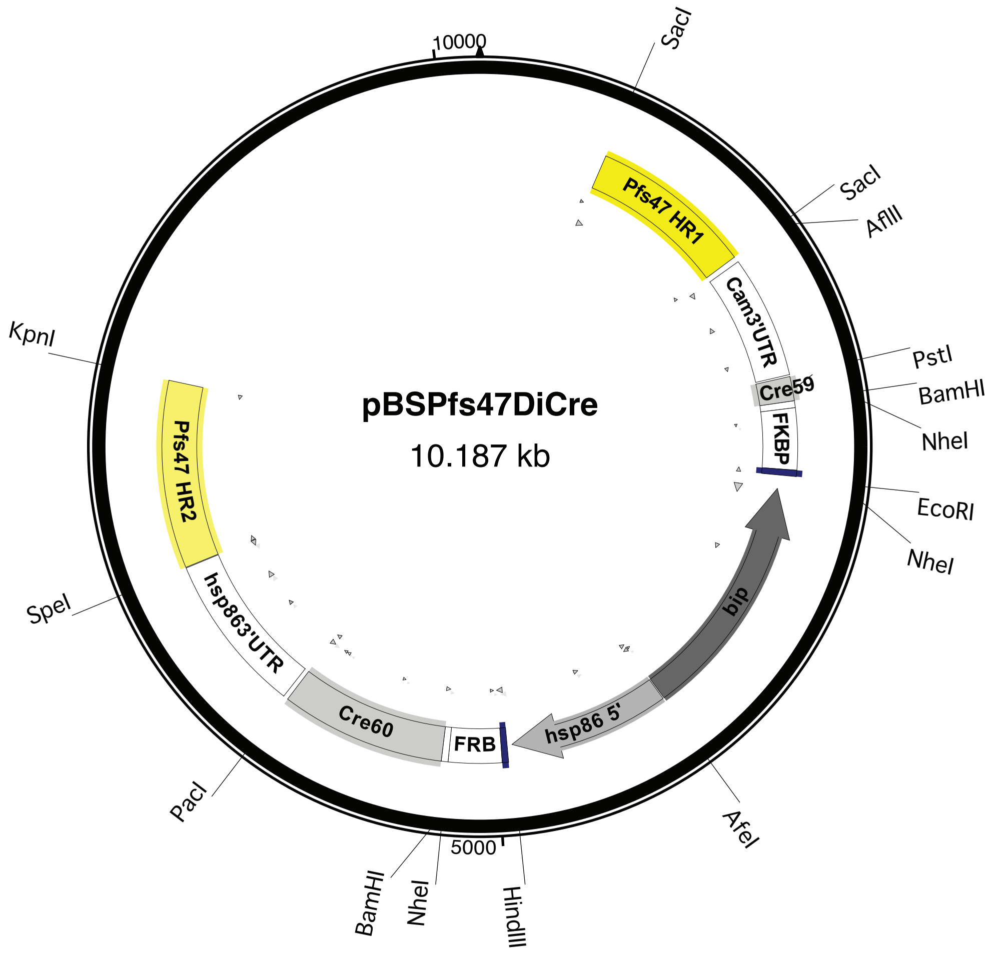

Supplementary Figure S3:

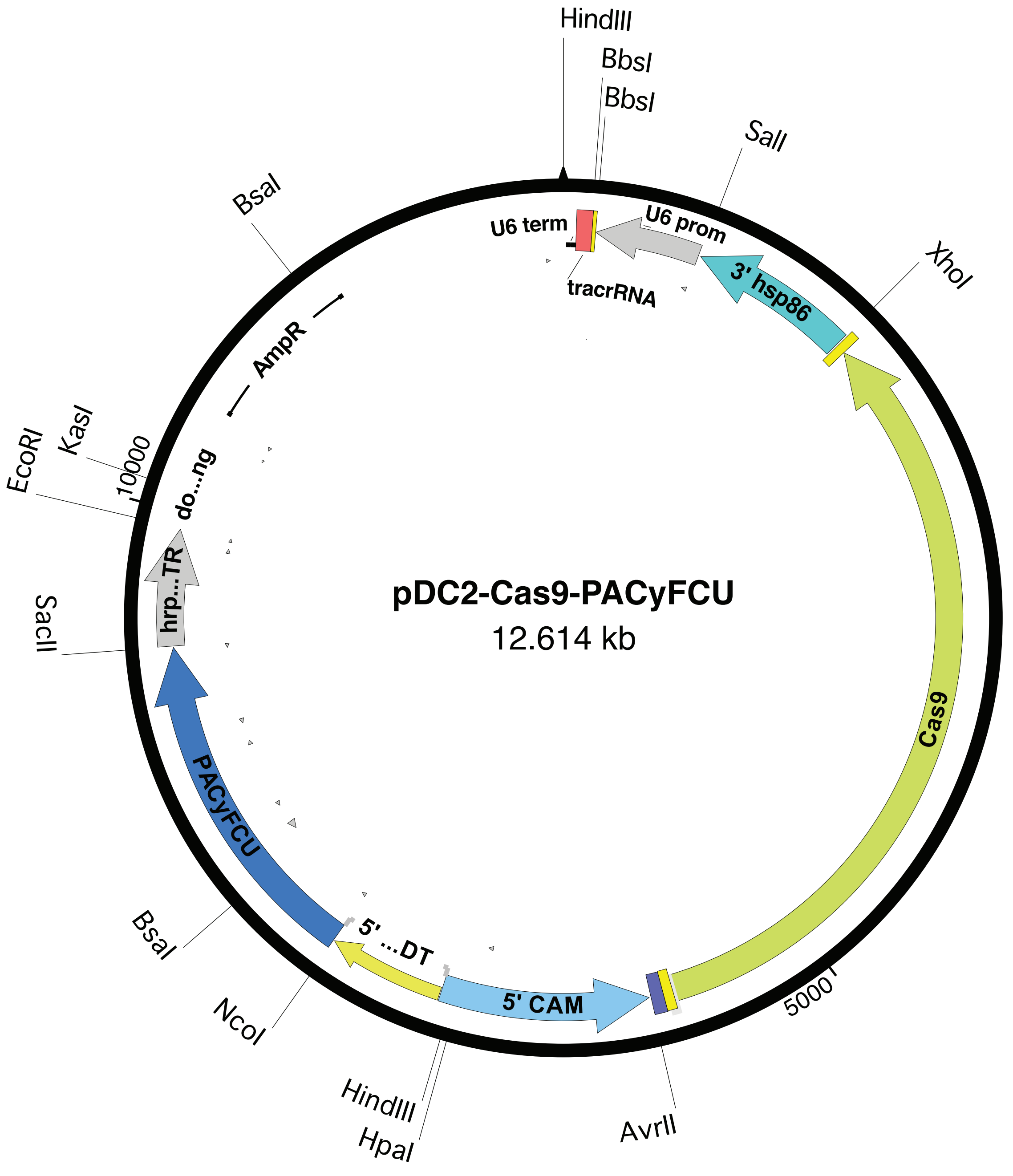

Supplementary Figure S4:

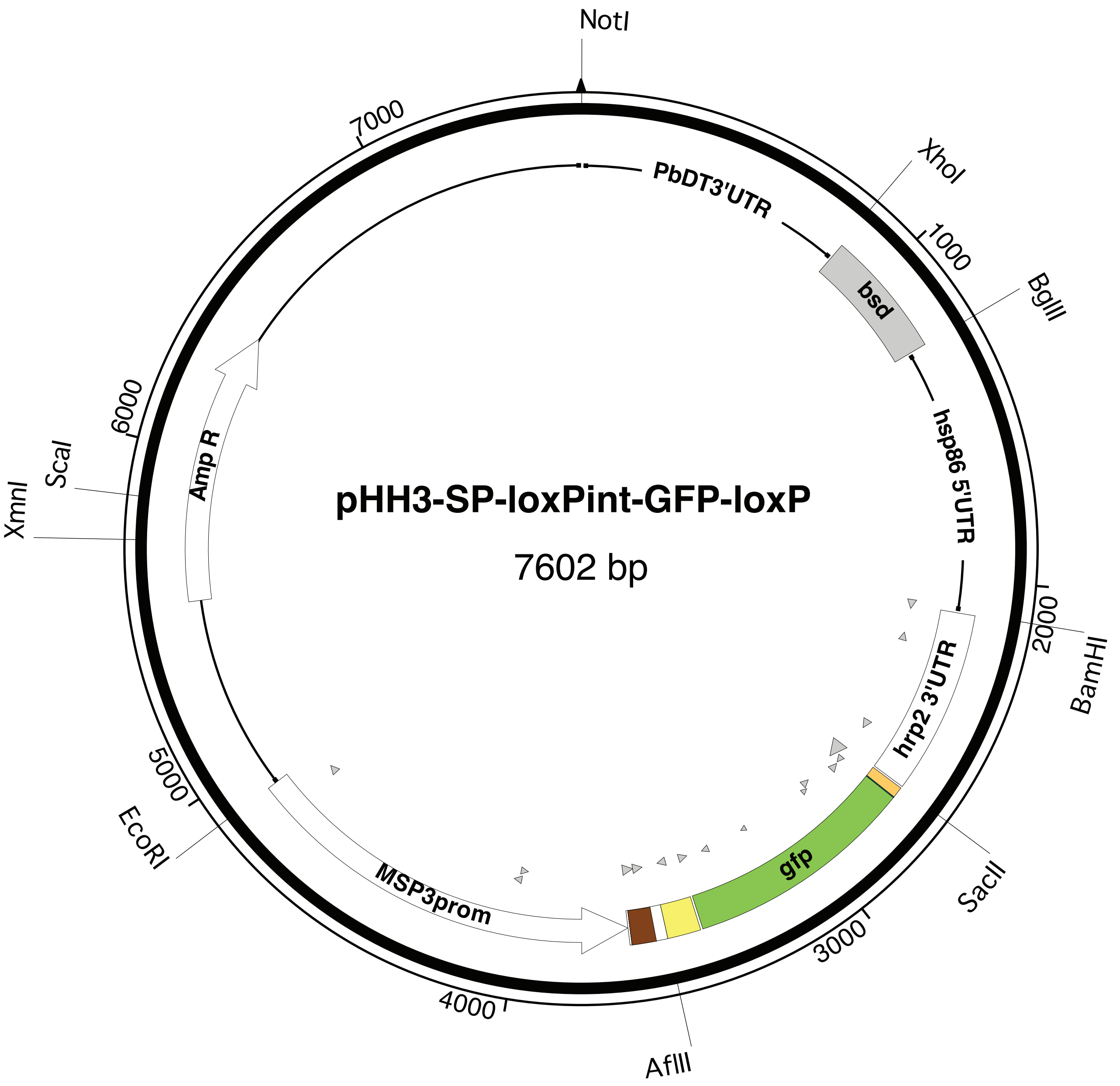

Supplementary Fig S5:

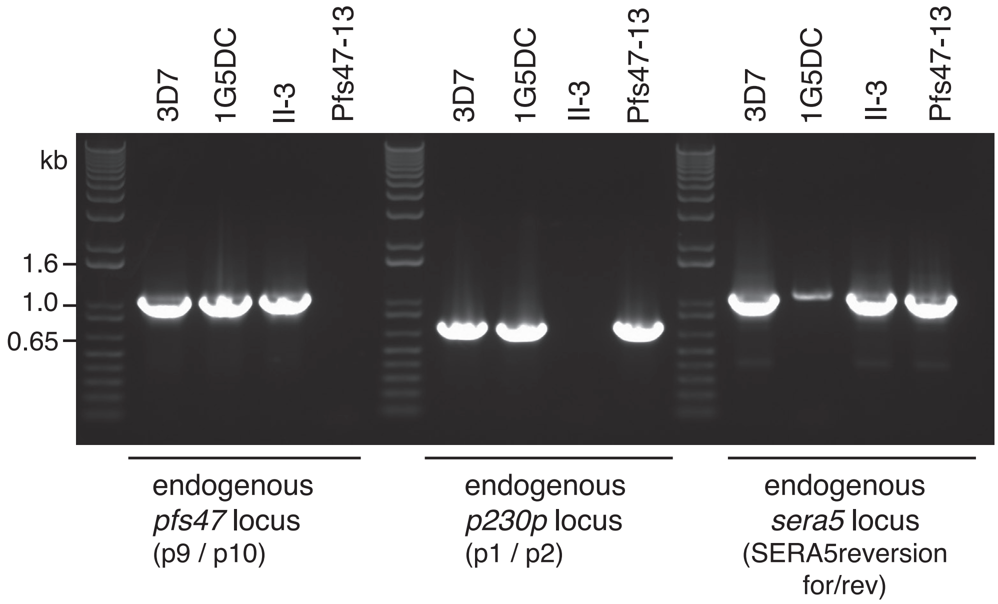

Supplement: Supplementary file 1 — Supplementary Information [file 41598_2017_3984_MOESM1_ESM.pdf]
